# Supplementary material for: Plant traits poorly predict winner and loser shrub species in a warming tundra biome
Source: Nat Commun. 2023 Jun 28;14:3837. doi: 10.1038/s41467-023-39573-4 (PMC10307830; doi:10.1038/s41467-023-39573-4)
Supplement: Supplementary file 1 — Supplementary Information [file 41467_2023_39573_MOESM1_ESM.pdf]

Supplementary Information to ‘Plant traits poorly predict winner and loser shrub species in a warming tundra biome’

Mariana García Criado*, Isla H. Myers-Smith, Anne D. Bjorkman, Signe Normand, Anne Blach-Overgaard, Haydn J.D. Thomas, Anu Eskelinen, Konsta Happonen, Juha M. Alatalo, Alba Anadon-Rosell, Isabelle Aubin, Mariska te Beest, Katlyn R. Betway-May, Daan Blok, Allan Buras, Bruno E.L. Cerabolini, Katherine Christie, Hans C. Cornelissen, Bruce C. Forbes, Esther R. Frei, Paul Grogan, Luise Hermanutz, Robert D. Hollister, James Hudson, Maitane Iturrate-Garcia, Elina Kaarlejärvi, Michael Kleyer, Laurent J. Lamarque, Jonas J. Lembrechts, Esther Lévesque, Miska Luoto, Petr Macek, Jeremy L. May, Janet S. Prevéy, Gabriela Schaepman-Strub, Serge N. Sheremetiev, Laura Siegwart Collier, Nadejda A. Soudzilovskaia, Andrew Trant, Susanna E. Venn and Anna-Maria Virkkala

* Corresponding author: mariana.garcia.criado@gmail.com

Supplementary Methods – Species Distribution Models (SDMs)

Supplementary methods on the original creation of SDMs whose outputs (current species range sizes and projected range shifts) were used for analyses in this study.

Species occurrence data

Species occurrence data was obtained from a variety of sources including published dot and range outline maps[1–5], and occurrence records extracted from the Global Biodiversity Information Facility (GBIF; http://www.gbif.org/). The taxonomy followed The Plant List (http://www.theplantlist.org/). Names of synonyms and sub-species for each species were obtained from the Annotated Checklist of the Panarctic Flora (PAF) to ensure to match the distribution data from the different sources to the correct species.

Processing the range maps

The Hultén maps from Hultén[4] were georeferenced and digitised in geographic information systems (GIS) following several steps in ArcGIS 10.2.2 (ESRI, Redlands, CA, USA). Initially, all original range map images were visually inspected to evaluate how many individual geographical regions the range outlines could be split into (e.g., North America, Greenland, Asia). For example, a species range consisting of multiple non overlapping areas covering both Greenland and North America were split into two geographical regions in the following processes. First, for each species the original Hultén map was georeferenced using the georeferencing tool in ArcGIS focussing on the selected region (e.g., Greenland) to match the range map to an underlying continental polygon layer in the Polar Lambert Azimuthal Equal Area projection. This was done separately for all regions identified for each map resulting in multiple georeferenced range maps subsets per species. Second, for each region, all previously digitized species ranges (polygon layers), which no longer matched the underlying original range map were geographically shifted using the link table generated during the georeferencing process to make up for the georeferencing mismatch so that distances and areas across the world became comparable. Polygons which still did not match the underlying ranges were subsequently shifted manually. All processes were repeated for all polygon layers per species per region. Third, the polygon layers representing region-specific parts of the species' range as well as region-specific layers for subspecies and synonyms where rasterized at 10x10 km resolution in the Polar Lambert Azimuthal Equal Area projection and summed to have one final rasterized range map for each species. The summed raster layers were subsequently reclassified to a binary map so areas where the shifted polygon layers overlapped were given the value 1 and all areas with no range information were classified as 0. The range maps of Hultén[4] only included North American ranges. These were georeferenced and digitised and geoprocessed in ArcGIS 10.2.2 to match the maps of Hultén & Fries (1986)[5]. The USGS maps (U.S. Geological Survey, 1999) were already available in polygons. These were projected to the Polar Lambert Azimuthal Equal Area projection and where rasterized at 10 x 10 km. For all range outlines, the latitude and longitude for each 10 × 10 km grid in which the species was present, were subsequently extracted.

Dot maps

Dot maps for species occurring in Greenland were georeferenced and digitized per region (north, west and south) according to the source images from three different publications[1–3] in the Polar Lambert Azimuthal Equal Area projection. The dots were digitised as points and the latitude and longitude for each occurrence were extracted and in this process merged the regional locality records per species.

GBIF data

Species records for all species and synonyms were downloaded through the GBIF data portal (http://www.gbif.org/). The raw GBIF tables were subsequently filtered to extract only the georeferenced occurrences with a specific precision level of the geographical coordinates smaller than or equal to 8,000 meters. The first step in the filtering process was to compute the precision level for each georeferenced record based on the number of decimal digits of the latitude and longitude and the position on the Earth using the Harvesine formula following Normand et al. (2013)[6]. In step two, spatially unique georeferences were extracted and subsequently records with precision levels above 8,000 metres were excluded from the dataset. In addition, occurrences with georeferences where both latitude and longitude were zero were also excluded from the analyses. A final step included the combination of occurrence data for species and their associated sub-species and synonyms and converting the latitude and longitude coordinates into the Polar Lambert Azimuthal Equal Area projection.

Presence and pseudo-absence data

For each species, data from the different sources were combined using the following procedure. First, to put more emphasis on known observations, records obtained from GBIF and the dot map sources were prioritised by excluding the range-outline derived records in the vicinity (50 km radius around each record) of any known occurrences. Secondly, to avoid issues with pseudo-replication and spatial autocorrelation, the spatial bias often evident in e.g., GBIF records due to biased sampling effort was reduced. From the full combined presence dataset ten different and reduced presence

datasets were extracted by randomly selecting a presence record from the full dataset and excluding any other presences in a radius of 50 km around this point. Subsequently, a new random presence was selected and the procedure was continued until no more presences were available to select. Then a new presence dataset was initialised by selecting a random presence and repeating the process until the resulting ten datasets were extracted.

For each of the ten presence datasets, a unique pseudo-absence dataset was extracted. An approach was chosen to avoid sampling pseudo-absences in regions where the species in question is unlikely to occur, as this could inflate the test statistics in the species distribution modelling[7]. Therefore, the pseudo-absence selection was restrained within a 500 km buffer around the thinned presences of the full dataset per species. The pseudo-absences were randomly selected with the following geographical constraints: Each point was selected at least 50 km apart and at least 50 km away from any given presence in the presence data subset. The sampling was terminated when no more pseudo-absences were available within the buffer or when 10,000 records were reached.

Climate data

Climatic data for the period 1950-2000 was used from the Worldclim dataset[8], here referred to as the baseline period. Future climate data was obtained for the end of the century (2070-2099, hereafter referred to as 2080) from the International Centre for Tropical Agriculture (CIAT) dataset of the Fifth Intergovernmental Panel on Climate Change report using the spatial downscaling data of the Delta Method[9]. To make sure the models covered a wide range of climatic futures, future climate data were selected from six different coupled Atmosphere-Ocean global circulation models (GCMs) for four different Representative Concentration Pathway (RCP) scenarios (RCP2.6, RCP4.5, RCP6.0, RCP8.5). The RCPs represent different assumptions about socio-economic, technological, demographic and institutional futures. In total, 24 future climate datasets were used (each combination of GCM and RCP). To assure that the future climate datasets represented a broad spatial variation in climate, GCMs with very different climate sensitivities were selected, ranging from 2.1-4.1°C, namely

cesm1-cam5, csiro-mk3.6.0, gfdl-esm2m, miroc-miroc5, mri-cgcm3, and nimr-hadgem2-ao.

For the baseline and the future climate datasets, ecologically meaningful climate variables for Arctic plants were selected, namely minimum temperature of the coldest month, summer temperature, summer precipitation, and winter precipitation as well as a growing-degree-day layer (ddeg). The latter was computed from 12 monthly average temperature layers from January to December in 1/10 degree*day above 0º C for the baseline period and for all future climate scenarios. All layers were projected to the Polar Lambert Azimuthal Equal Area projection at 10 × 10 km resolution.

Trait information

To be able to account for dispersal in the distribution modelling, a set of species traits was compiled. Data on different plant traits were extracted from different sources. The main trait information on growth form, plant height and seed mass was extracted from the TRY Plant Trait Database[10]. In addition, the trait data from TRY was supplemented with trait information on growth form, maximum height, seed mass and dispersal syndrome deducted from eFloras (http://www.efloras.org/index.aspx/) and Flora indicative, Kew Botanic Garden (http://apps.kew.org/floraindica/home.do). Two classes of heights (generative plant height and vegetative plant height) were selected from TRY and the following sub-traits/covariates were subsequently included: plant height generative (including plant height reproductive and height at inflorescence top) as well as plant height vegetative (including height at 20 years, average tree height, flowering plant height top leaf elongated, flowering plant height top leaf not elongated, vegetative height not elongated, plant height vegetative).

Most units in TRY were already standardised to meters, but "height at 20 years" was still in feet, so they were recalculated to meters (using 1 feet = 0.3048 meters). For seed mass, information was extracted on the class “seed dry mass” from TRY. For each sub-trait of height and for seed mass information for the following classes was extracted if available: Best estimate, High, Low, Maximum, Minimum, Mean, Median, Single and Site specific mean. Some of the classes, namely “low”, “high”, and “site specific mean” were assumed to represent “minimum”, “maximum”, and “mean”,

respectively and were changed accordingly. For each height trait (plant height generative and plant height vegetative) and seed mass, the median for each class (e.g., maximum) per species were computed (e.g., median of all Mean values, Best estimates, etc.). This resulted in a total of 11 height variables and five seed mass variables.

Trait information was not available for all species in the current study; hence, the gaps were filled computing the median genus-level values within the same functional group for a given species. If this was not available, family-level values within functional groups were used, and if that was not available, functional group values were used instead.

Migration rates

Species-specific migration rates were predicted to get an estimate of the maximum distance a given species will potentially spread per year for the analysis of species range dynamics (MIG). The dispersal rates were predicted using linear mixed models implemented in the DISPERSAL function of Tamme et al. (2014)[11]. This function uses information on the following species-specific traits: dispersal syndrome (DS), growth form (GF), seed mass (SM), and maximum height (MH) and predicts dispersal rate as DS + GF + SM + MH. Plant family was included as a random factor to account for the taxonomic hierarchy in the data, which was found in Tamme et al. (2014)[11] to provide more accurate models for modelling dispersal rates (see Tamme et al. 2014[11] for details). Species were assigned to family based on The Plant List (http://www.theplantlist.org/).

Species distribution modelling

To model the distribution of tundra shrub species, species distribution models were used (SDM) by applying six different algorithms (Maxent, Random Forest, Generalised Linear Models, Boosted Regression Tree, MARS and Classification Tree Analysis) implemented in the R package BIOMOD2. For most algorithms, the default settings of BIOMOD2 were used, however for some algorithms, specific settings were applied: for Maxent the selection of 10,000 background points from the entire study area were allowed, and only the linear, quadratic and hinge features were selected. For GBM, a

maximum of 1,000 trees were run, and for GLM the polynomial function and first level interactions were chosen. To evaluate the performance of the models, each of the ten presence data sets was split in two: 80% of the data were used for calibrations and 20% for testing. Random splitting was repeated 10 times and model performance assessed using True Skilled Statistics (TSS) and the Area Under the Receiver operating Curve (AUC). Hence, for each species, 660 models across six SDM algorithms were constructed using ten different presence/pseudo-absence data sets each split randomly 10 times as well as a final full model (including all data points). All models for a species were combined in ensemble models using the coefficient of variation of probabilities, median of probabilities, models committee averaging, as well as the weighted mean of probabilities implemented in BIOMOD2. For all ensembles, only models with TSS scores > 0.5 were allowed to enter the final ensemble model. The ensemble models were evaluated using TSS and AUC. Based on the evaluation of the ensemble models, the model committee averaging was selected as this provided the overall best modelling results in terms of statistical power compared to single algorithms and the other ensemble models[12]. Models were considered to be good if the TSS ranged from 0.7 to 1. In this way, TSS values ranging between 0.7 and 1.0 were not distinguished and thus avoided the selection of overfitted models.

Models, dispersal scenarios, and projections

To estimate the species ranges for the baseline period (present day), a pure climatic model (CLIM) was constructed using climate data for the baseline period in combination with the occurrence and pseudo-absence data for each species. To estimate future potential ranges for all species, the species-specific CLIM models were projected onto future climates for all combinations of RCPs and GCMs (‘unlimited dispersal’). To make realistic estimations of species potential range shifts, a prediction was made on how far a species could potentially disperse in 100 years from their current range margins (‘limited dispersal’). To do this, species-specific trait-based migration rates (MIG) were applied as described below.

To compute range shifts over time, a realistic representation of each species’ current range was needed. As species are unlikely to occur at all climatically suitable locations, the CLIM baseline projections were subsequently constrained with a

species-specific mask based on a convex hull around all presences with an added 250 km buffer. The continuous suitability prediction for the baseline period of the spatially constrained CLIM model to binary predictions was converted by using the thresholds which maximise TSS. The boundary of the final binary output was used as an estimation of a given species' actual range.

The future projections of the CLIM models were filtered using the predicted migration rates, which are termed the 'limited dispersal' scenario, in accordance to the time range used in the current study (baseline: 1950-2000, future: 1970-2100). The upper boundary of the confidence interval of the linear mixed models was used[11] as the maximum dispersal distances per year for a given species based on the DISPERSAL function. The dispersal rate (meters/year) was multiplied by 100 to the range margin of the actual range and only considered the grids falling within this region for any further processing of all future projections. Continuous suitability projections were also converted to binary outputs using the thresholds which maximises TSS. For comparison to the 'limited dispersal' scenario, the potential range shifts for a 'no dispersal' and an 'unlimited dispersal' scenario were also computed. For the 'no dispersal' scenario, all future projections were limited within the boundary of a given species' estimated actual range. For the 'unlimited dispersal' scenario, all species were allowed to migrate to all climatically suitable sites across continents, i.e., these future projections were not limited by any set boundaries.

All data processing and analyses were conducted in R version 3.2.3 unless specified otherwise. The BIOMOD2 package version 2 3.1-73-05[13] was used and run in R version 3.2.0. The DISPERSAL function[11] was run in R version 3.2.3[14].

Supplementary Methods – Use of traits on model projections

From the three calculated dispersal scenarios ('no dispersal', 'limited dispersal' and 'unlimited dispersal'), we chose 'limited dispersal' range shifts for our analyses because it represents the most plausible future migration patterns for tundra plants. A 'no dispersal' scenario (corresponding here to the boundaries of a species' actual range) is considered to be less realistic as some amount of dispersal is expected at

plant range edges[6]. Likewise, an ‘unlimited’ dispersal scenario (allowing species to spread unlimited across all continents despite any potential spatial or time constraints) would be unrealistic and would at best only be a representation of the full climatic niche in geographical space with clear risk of over-estimating species future ranges. This means that for example, North American endemic species could be present in the Russian Arctic if climatic conditions are similar, which would likely substantially over-estimate range sizes (Figure S2). Therefore, we carried the analyses in this study using the ‘limited dispersal’ scenario which incorporates geographical constraints and better represents the real-world movements of species.

Four traits were incorporated into SDMs to define coarse average migration-specific rates in the ‘limited dispersal’ scenario (see ‘Trait information’ section above). Two of these traits were seed mass and plant height, meaning that there could be a potential circularity in modelling SDM-derived range shifts with seed mass and height trait values and variation. Therefore, we carried out additional analyses to understand whether the inclusion of seed mass and height records to calculate migration rates influenced our analyses.

First, we compared the ‘unlimited dispersal’ and ‘limited dispersal’ scenarios through univariate non-weighted linear regressions of the median absolute range shift versus seed mass values and height values. We found that seed mass in the ‘limited scenario’ explained 0.01% more of the projected range shifts than the ‘unlimited scenario’ (R^2 = 0.05 versus R^2 = 0.04, respectively). Similarly, plant height in the ‘limited scenario’ only explained 2% more of the projected range shifts than the ‘unlimited dispersal’ scenario (R^2 = 0.044 versus R^2 = 0.024, respectively). Second, we modelled the range shifts versus trait values and variation models (as in Figure 5) with the range shift values from the ‘unlimited scenario’ for comparison. We found similar findings to the ‘limited dispersal’ scenario, with no significant relationships between median absolute range shifts and trait values nor variation (Figure 5, Figure S2). Therefore, we conclude that the potential circularity in using the ‘limited dispersal’ scenario does not influence the main findings of our study.

Online sources of categorical traits and maximum height

All sources accessed during June 2020.

US Forest Service: https://www.fs.usda.gov/

United States Department of Agriculture: https://www.usda.gov/

Flora of North America: http://www.efloras.org/flora_page.aspx?flora_id=1

Royal Horticultural Society: https://www.rhs.org.uk/

Missouri Botanical Garden: https://www.missouribotanicalgarden.org/

Atlas of the British and Irish Flora: https://www.brc.ac.uk/plantatlas/

LEDA database: https://uol.de/en/landeco/research/leda

List of published datasets within the downloaded TRY database

Atkin, O. K. *et al.* Global variability in leaf respiration among plant functional types in relation to climate and leaf traits. *New Phytologist* doi:10.1111/nph.13253 (2015).

Aubin, I. *et al.* TOPIC–traits of plants in Canada. Natural Resources Canada, Canadian Forest Service, Sault Ste. Marie, Ontario. Online [URL] TOPIC website: http://cfs.cloud.nrcan.gc.ca/ctn/topic.php (2012).

Aubin, I. *et al.* Traits to stay, traits to move: a review of functional traits to assess sensitivity and adaptive capacity of temperate and boreal trees to climate change. *Environmental Reviews* **24**, 164-186 doi:10.1139/er-2015-0072 (2016).

Bahn, M. *et al.* Leaf photosynthesis, nitrogen contents and specific leaf area of 30 grassland species in differently managed mountain ecosystems in the Eastern Alps. Pages 247-255 in A. Cernusca, U. Tappeiner, and N. Bayfield, editors. *Land-use changes in European mountain ecosystems.* ECOMONT- Concept and Results. Blackwell Wissenschaft, Berlin (1999).

Blonder, B. *et al.* The shrinkage effect biases estimates of paleoclimate. *American Journal of Botany* **99.11**, 1756-1763 (2012).

Bond-Lamberty, B., Wang, C. & Gower, S. T. Leaf area dynamics of a boreal black spruce fire chronosequence, *Tree Physiology* **22**, 993-1001 (2002).

Cerabolini, B., Pierce, S., Luzzaro, A. & Ossola A. Species evenness affects ecosystem processes in situ via diversity in the adaptive strategies of dominant species. *Plant Ecology*, **207**, 333-345 (2010).

Cerabolini, B.E.L. *et al.* Can CSR classification be generally applied outside Britain? *Plant Ecology* **210**, 253-261 (2010).

Ciocarlan, V. The illustrated Flora of Romania. Pteridophyta et Spermatopyta. Editura Ceres, 1141 pp (in Romanian) (2003).

Sanda, V., Bita-Nicolae, C. D. & Barabas, N. The flora of spontane and cultivated cormophytes from Romania. Editura “Ion Borcea”, Bacau, 316 pp (in Romanian) (2009).

Cornelissen, J. H. C. An experimental comparison of leaf decomposition rates in a wide range of temperate plant species and types. *Journal of Ecology* **84**, 573-582 (1996).

Cornelissen, J. H. C., *et al.* Functional traits of woody plants: correspondence of species rankings between field adults and laboratory-grown seedlings? *Journal of Vegetation Science* **14**, 311-322 (2003).

Cornelissen, J. H. C. *et al.* Leaf digestibility and litter decomposability are related in a wide range of subarctic plant species and types. *Functional Ecology* **18**, 779-786 (2004).

Cornelissen, J. H. C., Werger, M. J. A., CastroDiez, P., vanRheenen, J. W. A. and Rowland, A. P. Foliar nutrients in relation to growth, allocation and leaf traits in

seedlings of a wide range of woody plant species and types. *Oecologia* **111**, 460-469 (1997).

Cornelissen, J. H. C., Diez, P. C. and Hunt, R. Seedling growth, allocation and leaf attributes in a wide range of woody plant species and types. *Journal of Ecology* **84**, 755-765 (1996).

Cornelissen, J. H. C. *et al.* Leaf structure and defence control litter decomposition rate across species and life forms in regional floras on two continents. *New Phytologist* **143**, 191-200 (1999).

Cornelissen, J. H. C., Diez, P. C. & Hunt, R. Seedling growth, allocation and leaf attributes in a wide range of woody plant species and types. *Journal of Ecology* **84**, 755-765 (1996).

Cornelissen, J. H. C., Aerts, R., Cerabolini, B., Werger, M. J. A. & van der Heijden, M. G. A. Carbon cycling traits of plant species are linked with mycorrhizal strategy. *Oecologia* **129**, 611-619 (2001).

Fitter, A. H. and Peat, H. J. The Ecological Flora Database. *Journal of Ecology* **82**, 415-425 (1994).

Freschet, G. T., Cornelissen, J. H. C., van Logtestijn, R. S. P. & Aerts, R. Evidence of the ‘plant economics spectrum’ in a subarctic flora. *Journal of Ecology* **98**, 362-373 (2010).

Freschet, G. T., Cornelissen, J. H. C., van Logtestijn, R. S. P. & Aerts, R. Substantial nutrient resorption from leaves, stems and roots in a sub-arctic flora: what is the link with other resource economics traits? *New Phytologist* **186**, 879-889 (2010).

Garnier, E. *et al.* Assessing the effects of land-use change on plant traits, communities and ecosystem functioning in grasslands: A standardized methodology and lessons from an application to 11 European sites. *Annals of Botany* **99**, 967-985. (2007).

Hattermann, D., Elstner, C., Bernhardt-Römermann, M. & Eckstein, L. Measurements from the project “Relative effects of local and regional factors as drivers for plant community diversity, functional trait diversity and genetic structure of species on Baltic uplift islands” funded by the German Research Foundation - DFG: BE 4143/5-1 and EC 209/12-1.

Kattge, J., Knorr, W., Raddatz, T., & Wirth, C. Quantifying photosynthetic capacity and its relationship to leaf nitrogen content for global-scale terrestrial biosphere models. *Global Change Biology* **15**, 976-991 (2009).

Laughlin, D. C., Leppert, J. J. Moore, M. M. & Sieg, C. H. A multi-trait test of the leaf-height-seed plant strategy scheme with 133 species from a pine forest flora. *Functional Ecology* **24**, 493-501 (2010).

Laughlin, D. C., Fulé, P.Z., Huffman, D.W., Crouse, J. & Laliberte, E. Climatic constraints on trait-based forest assembly. *Journal of Ecology* **99**, 1489-1499 (2011).

Maire, V. *et al.* Global soil and climate effects on leaf photosynthetic traits and rates. *Global Ecology and Biogeography* **24,** 706-717 (2015).

Maire, V. *et al.* Data from: Global effects of soil and climate on leaf photosynthetic traits and rates. Dryad Digital Repository. http://dx.doi.org/10.5061/dryad.j42m7 (2015)

Milla, R. & Reich, P. B. Multi-trait interactions, not phylogeny, fine-tune leaf size reduction with increasing altitude. *Annals of Botany* **107**, 455–465 (2011).

Moles, A. T. *et al.* Factors that shape seed mass evolution. *Proceedings of the National Academy of Sciences of the United States of America* **102**, 10540-10544 (2005).

Moles, A. T., Falster, D. S., Leishman, M. R. & Westoby, M. Small-seeded species produce more seeds per square metre of canopy per year, but not per individual per lifetime. *Journal of Ecology* **92**, 384-396 (2004).

Moles, A.T. *et al.* Global patterns in plant height. *Journal of Ecology* **97**, 923-932 (2009).

Mori, A. S. *et al.* Functional redundancy of multiple forest taxa along an elevational gradient: predicting the consequences of non-random species loss. *Journal of Biogeography* **42**, 1383–1396. doi:10.1111/jbi.12514 (2015).

Niinemets, U. Components of leaf dry mass per area - thickness and density - alter leaf photosynthetic capacity in reverse directions in woody plants. *New Phytologist* **144**, 35-47 (1999).

Niinemets, U. Global-scale climatic controls of leaf dry mass per area, density, and thickness in trees and shrubs. *Ecology* **82**, 453-469 (2001).

Onoda, Y. *et al.* Global patterns of leaf mechanical properties. *Ecology Letters* **14**, 301-312 (2011).

Ordonez, J. C. *et al*. Leaf habit and woodiness regulate different leaf economy traits at a given nutrient supply. *Ecology* **91**, 3218-3228 (2010).

Ordonez, J. C. *et al*. Plant Strategies in Relation to Resource Supply in Mesic to Wet Environments: Does Theory Mirror Nature? *American Naturalist* **175**, 225-239 (2010).

Pierce, S., Brusa, G., Vagge, I. & Cerabolini, B. E. L. Allocating CSR plant functional types: the use of leaf economics and size traits to classify woody and herbaceous vascular plants. *Functional Ecology*, **27**, 1002-1010 (2013).

Pierce, S., Ceriani, R.M., De Andreis, R., Luzzaro, A. & Cerabolini, B. The leaf economics spectrum of Poaceae reflects variation in survival strategies. *Plant Biosystems* **141**, 337-343 (2007).

Pierce, S., Luzzaro, A., Caccianiga, M., Ceriani, R.M. & Cerabolini, B. Disturbance is the principal ?-scale filter determining niche differentiation, coexistence and biodiversity in an alpine community. *Journal of Ecology* **95**, 698-706 (2007).

Prentice, I.C. *et al.* Evidence for a universal scaling relationship of leaf CO_2 drawdown along a moisture gradient. *New Phytologist* **190,** 169–180 (2011).

Quested, H. M. *et al.* Decomposition of sub-arctic plants with differing nitrogen economies: A functional role for hemiparasites. *Ecology* **84**, 3209-3221 (2003).

Reich, P. B., *et al.* Scaling of respiration to nitrogen in leaves, stems and roots of higher land plants. *Ecology Letters* **11,** 793-801 (2008).

Rogers, A., Serbin, S. P., Ely, K. S., Sloan, V. L. & Wullschleger, S. D. Terrestrial biosphere models underestimate photosynthetic capacity and CO_2 assimilation in the Arctic. *New Phytologist* doi: 10.1111/nph.14740 (2017).

Schweingruber, F.H. & Landolt, W. The Xylem Database. Swiss Federal Research Institute WSL. (2005).

Schweingruber, F.H. & Poschlod, P. Growth rings in herbs and shrubs: Life span, age determination and stem anatomy. *Forest, Snow and Landscape Research* **79**, 195-415 (2005).

Shipley, B., Trade-offs between net assimilation rate and specific leaf area in determining relative growth rate: relationship with daily irradiance, *Functional Ecology* **16**, 682-689 (2002).

Shipley, B. The Use of above-Ground Maximum Relative Growth-Rate as an Accurate Predictor of Whole-Plant Maximum Relative Growth-Rate. *Functional Ecology* **3**, 771-775 (1989).

Shipley, B. Structured Interspecific Determinants of Specific Leaf-Area in 34 Species of Herbaceous Angiosperms. *Functional Ecology* **9**, 312-319 (1995).

Shipley, B. & Lechowicz, M. J. The functional co-ordination of leaf morphology, nitrogen concentration, and gas exchange in 40 wetland species. *Ecoscience* **7**, 183-194 (2000).

Shipley, B. & Parent, M. Germination Responses of 64 Wetland Species in Relation to Seed Size, Minimum Time to Reproduction and Seedling Relative Growth-Rate. *Functional Ecology* **5**, 111-118 (1991).

Sodhi, D. S., Livingstone, S. W., Carboni, M. & Cadotte, M. W. Plant invasion alters trait composition and diversity across habitats. *Ecology and Evolution.* **9**, 6199– 6210 https://doi.org/10.1002/ece3.5130 (2009).

Thuiller, W. Traits of European Alpine Flora - OriginAlps Project. Centre National de la Recherche Scientifique.

Vergutz, L., Manzoni, S., Porporato, A., Novais, R. F. & Jackson, R. B. Global resorption efficiencies and concentrations of carbon and nutrients in leaves of terrestrial plants. *Ecological Monographs* **82**, 205-220. doi: 10.1890/11-0416.1 (2012).

Vergutz, L., Manzoni, S., Porporato, A., Novais, R. F. & Jackson, R. B. A Global Database of Carbon and Nutrient Concentrations of Green and Senesced Leaves. Data set. Available on-line [http://daac.ornl.gov] from Oak Ridge National Laboratory

Distributed Active Archive Center, Oak Ridge, Tennessee, U.S.A. http://dx.doi.org/10.3334/ORNLDAAC/1106 (2012).

Walker, A. P. *et al.* The relationship of leaf photosynthetic traits – Vcmax and Jmax – to leaf nitrogen, leaf phosphorus, and specific leaf area: a meta-analysis and modeling study. *Ecology and Evolution* doi: 10.1002/ece3.1173 (2014).

Walker, A. P. A Global Data Set of Leaf Photosynthetic Rates, Leaf N and P, and Specific Leaf Area. Data set. Available on-line [http://daac.ornl.gov] from Oak Ridge National Laboratory Distributed Active Archive Center, Oak Ridge, Tennessee, USA. http://dx.doi.org/10.3334/ORNLDAAC/1224 (2014).

Wang, H. The China Plant Trait Database. PANGAEA, https://doi.org/10.1594/PANGAEA.871819 (2017).

Wirth, C. & Lichstein, J. W. The Imprint of Species Turnover on Old-Growth Forest Carbon Balances - Insights From a Trait-Based Model of Forest Dynamics. Pages 81-113 in C. Wirth, G. Gleixner, and M. Heimann, editors. Old-Growth Forests: Function, Fate and Value. Springer, New York, Berlin, Heidelberg (2009).

Wohlfahrt, G. *et al.* Inter-specific variation of the biochemical limitation to photosynthesis and related leaf traits of 30 species from mountain grassland ecosystems under different land use. *Plant, Cell and Environment* **22**, 1281-1296 (1999).

Wright, I. J. *et al.* The worldwide leaf economics spectrum. *Nature* **428**, 821-827 (2004).

Wright, I. J., *et al.* Irradiance, temperature and rainfall influence leaf dark respiration in woody plants: evidence from comparisons across 20 sites. *New Phytologist* **169**, 309-319 (2006).

Supplementary Figures and Tables

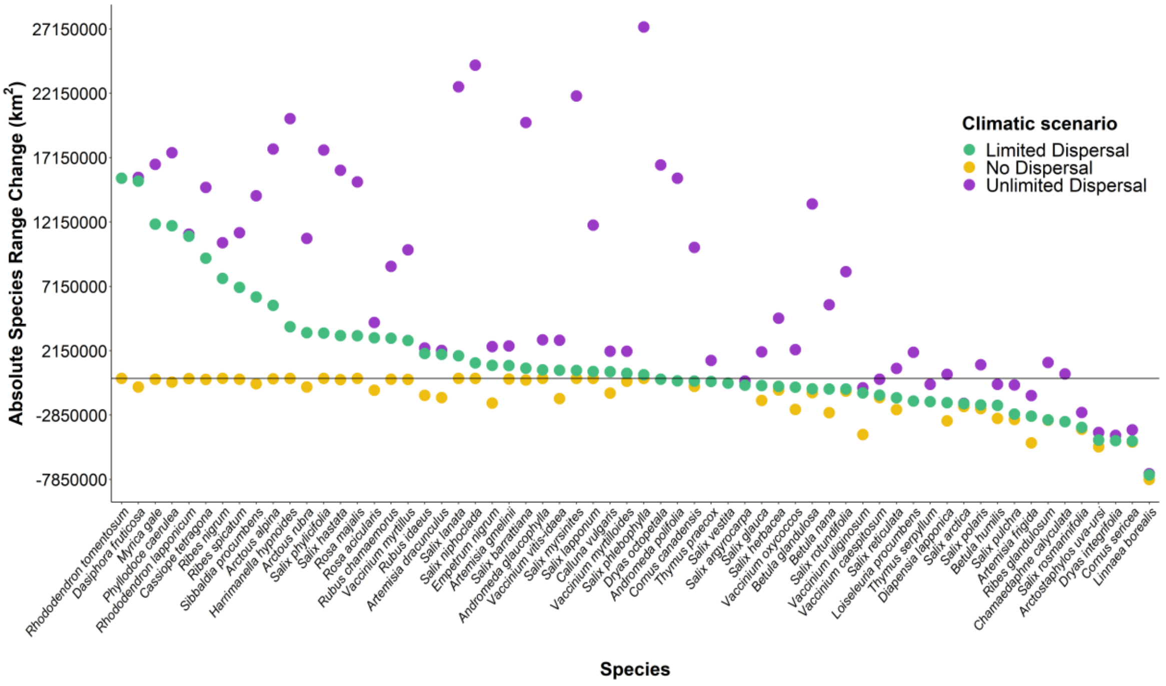

Figure S1. Representation of the median range shift value (across all 24 climatic scenarios) of the three climatic scenarios defined by dispersal per tundra shrub species. Absolute species range shifts were much larger for the 'unlimited dispersal' scenario, and 'limited dispersal' is considered to be the most realistic scenario from projections. The horizontal line represents a zero range shift.

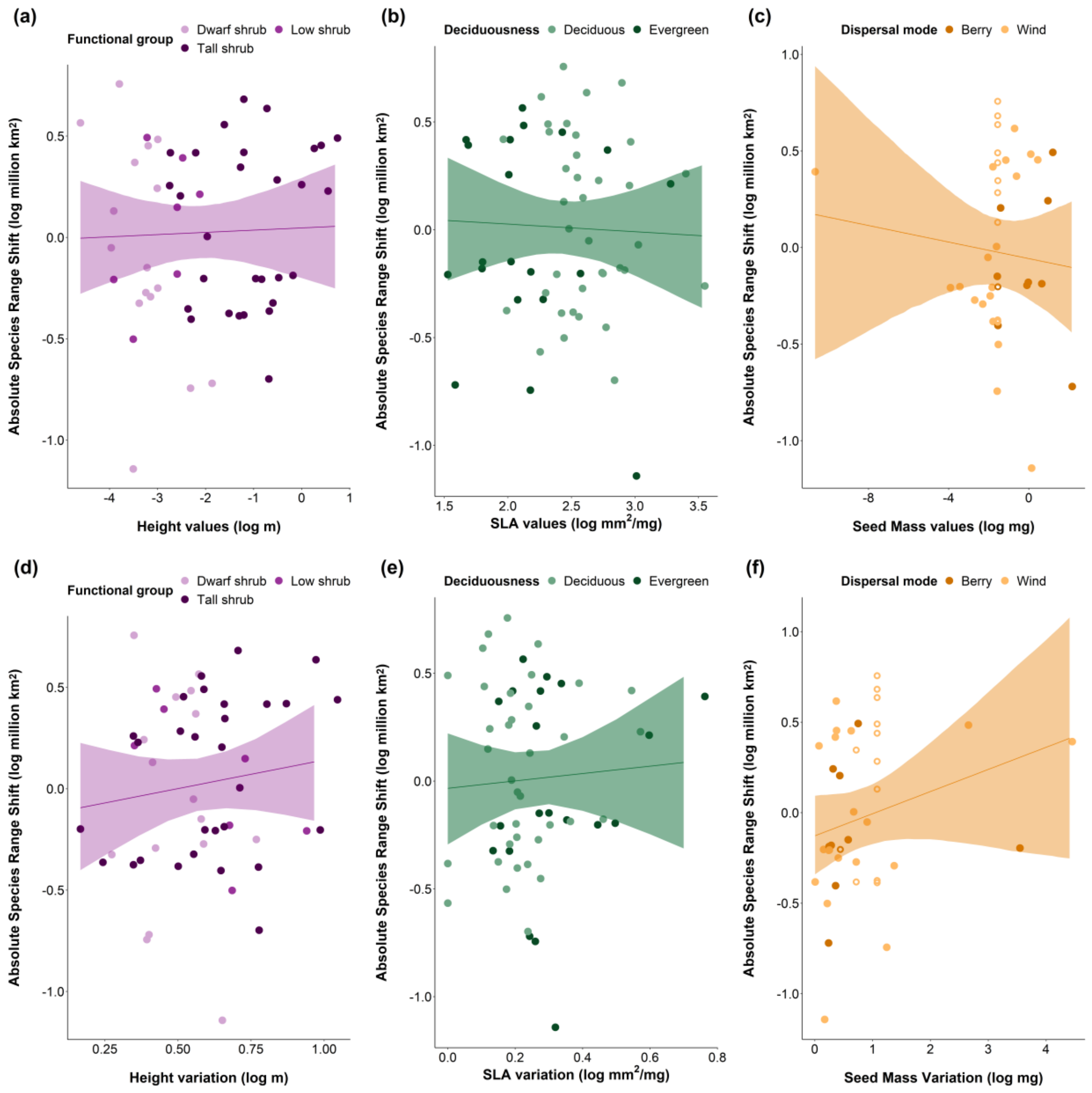

Figure S2. There were no clear relationships between individual trait values or variation and projected range shifts when using the 'unlimited dispersal' climatic scenario for tundra shrubs. Model outputs of the weighted linear regressions of projected range shifts as a function of **a)** height values, **b)** SLA values, **c)** seed mass values, **d)** height variation, **e)** SLA variation and **f)** seed mass variation. Trait values are the median per species and variation is the standard deviation of trait records. Points are raw values and coloured according to categorical traits related to each continuous trait. Lines are the predicted model slopes and the semi-transparent ribbons represent the 95% model credible intervals. Open circles in **c)** and **f)** represent the gap-filled seed mass values.

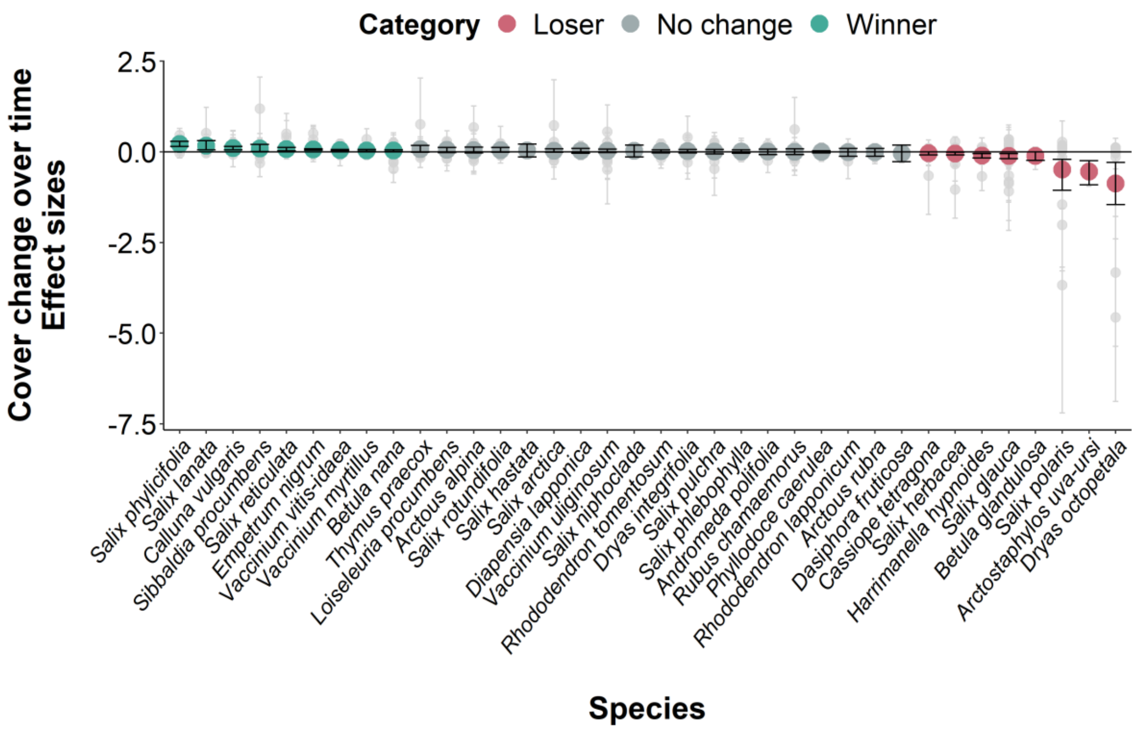

Figure S3. Slopes of models of cover change over time as per the ITEX database, measured as percent cover change per year for each tundra shrub species (n = 493 species slopes across subsites). Repeat surveys differed per sites, but were conducted over a minimum duration of five and up to 21 years (mean duration = 13.6 years) between 1989 and 2015. Species are ordered per mean slope value of cover change over time. Each light grey point represents the slopes per species and subsite, with their 95% credible intervals as error bars. Coloured points represent the overall slope per species across all plots and subsites, according to their category (winner, no change, loser), and error bars represent the 95% credible intervals. The horizontal line represents a cover change over time value of zero. For the purposes of visualization, a subsite point has been excluded from the graph: *Dryas octopetala* with a slope of -12.63 (CI = -18.77 to -1.76).

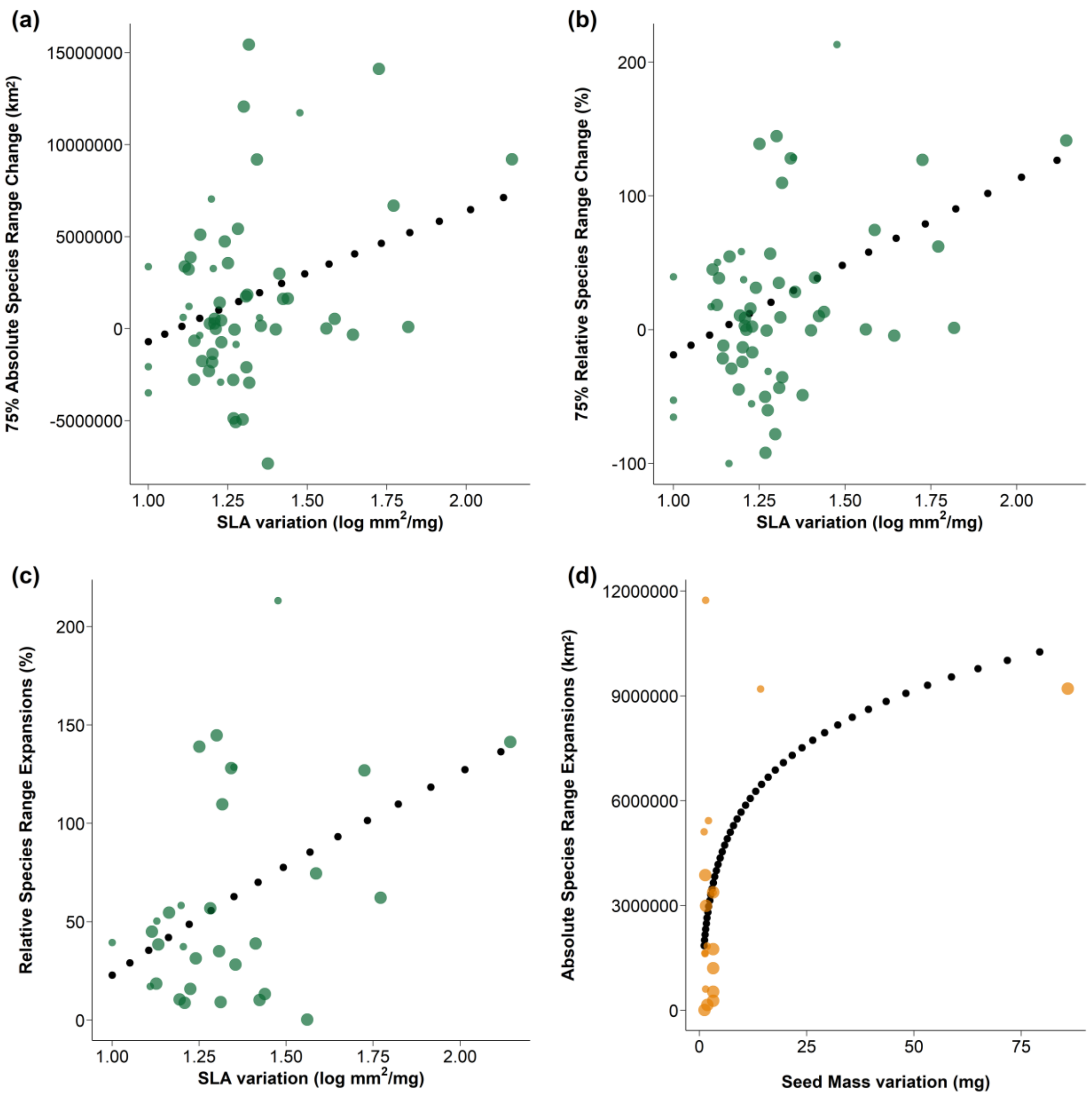

Figure S4. Selection of significant univariate models with back-transformed variables in their original units. Black dots are the predicted values from the models. Coloured points represent raw values. Smaller coloured points represent particular tundra shrub species that were down-weighted due to a small trait sample size.

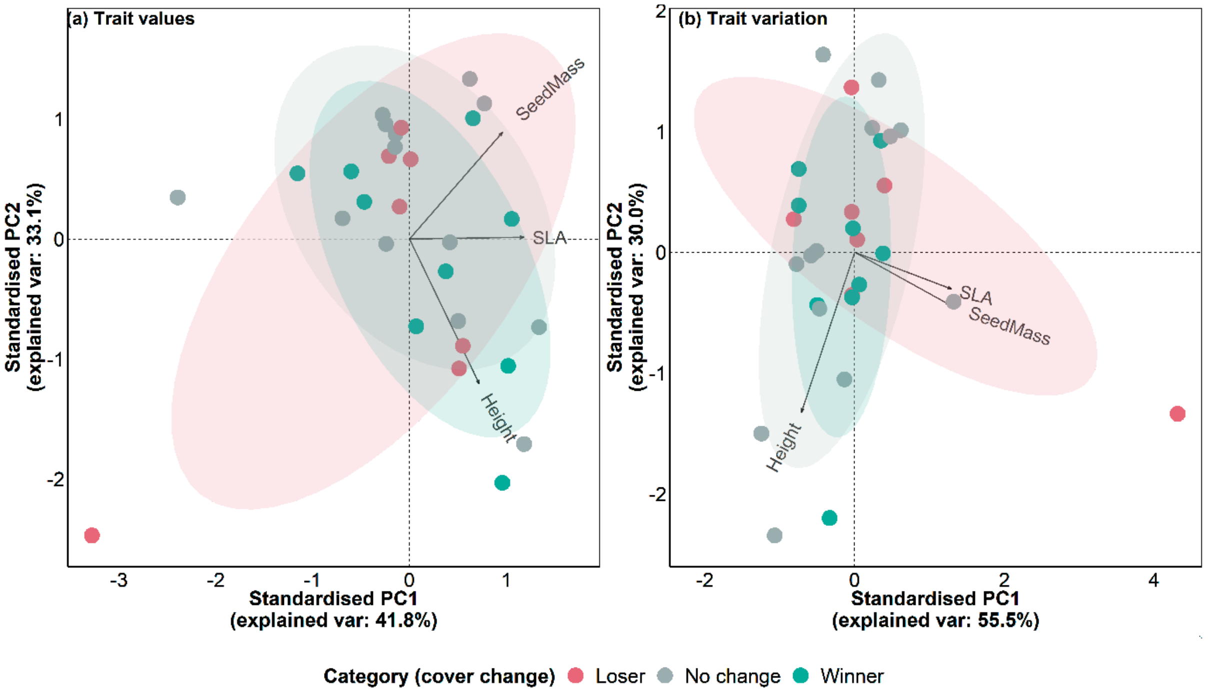

Figure S5. Principal Component Analysis for **a)** trait values and **b)** trait variation (*n* = 29) of tundra shrubs. Ellipses and points are coloured according to species categories based on cover change over time. Arrows indicate direction and weighting of each trait. Ellipses indicate the 68% confidence interval of distributions per category. **a)** For trait values, PC1 is driven mostly by seed mass and SLA, while PC2 is driven almost entirely by plant height. **b)** For trait variation, PC1 is driven by SLA and seed mass, and PC2 mostly by height.

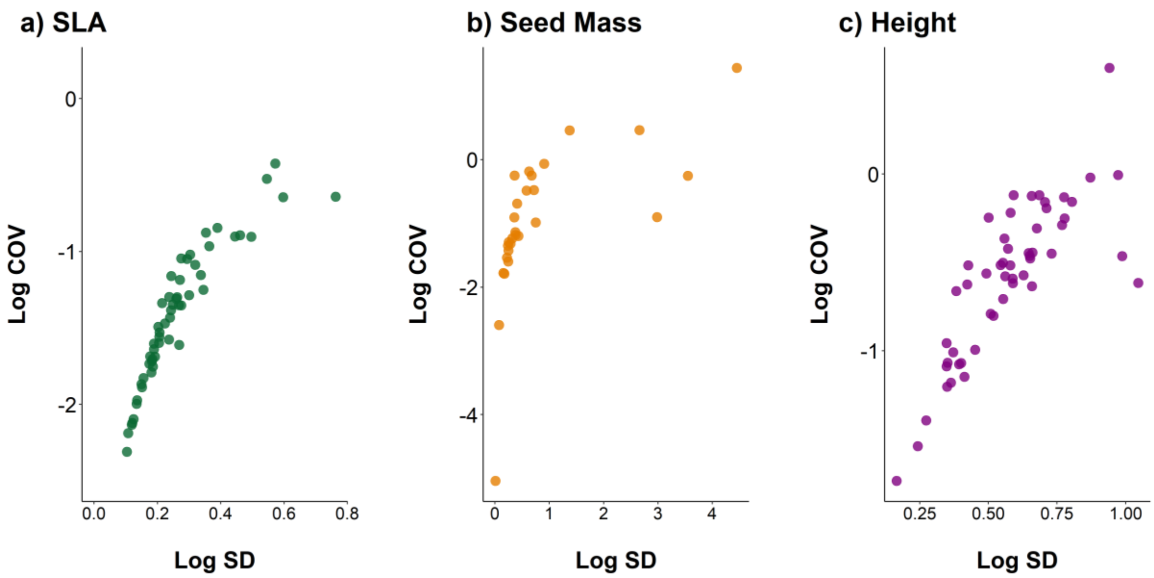

Figure S6. Log-transformed values of standard deviation (SD) and coefficient of variation (COV) are proportional for tundra shrubs. COV is calculated as SD/mean per species. Points represent the ITV value per species and are coloured according to each trait. The SLA ITV values of *Salix rosmarinifolia*, *Betula humilis* and *Salix phylicifolia* were calculated based on five records each of the same magnitude, and thus show no overall variance. Since their raw values are virtually zero, their log-transformed COV values are large (-28.2, -24.9 and -24.6, respectively), and thus these three outlier values are removed from the SLA plot for visualization purposes.

Supplementary Table 1. Summary statistics of the gap-filling procedure, outlining the number of gap-filled tundra shrub species per genus and the number of trait records, species and sites used to calculate gap-filled seed mass values. The two gap-filled *Betula* species share very similar traits to *B. nana*, so we consider this a representative sample even if only based on one sibling species.

Gap-filled genus	Number of gap-filled species	Number of trait records used to gap-fill	Number of species used to gap-fill	Number of sites used to gap-fill
Betula	2	6	1	3
Salix	9	40	4	10
Vaccinium	1	11	3	5

Supplementary Table 2. Classification of the species included in this study, ordered by decreasing absolute range change. We categorised species as 'winners' (if the 25% quantile of range change was above zero), 'no change' (if any quantile overlapped zero) or 'losers' (if the 75% quantile was below zero). We compare these categories with the ITEX cover change categories derived from Bjorkman et al.[15], where 'no change' species had 95% credible intervals that overlap zero, and 'winner' and 'loser' species slopes did not.

**Species**	**Median range change (absolute, km²)**	**Median range change (relative, %)**	**Functional Group**	**SDM-Projected Category**	**ITEX Cover Change Category**
Rhododendron tomentosum	15543250	110.4895	Tall shrub	Winner	No change
Dasiphora fruticosa	15327650	137.7815	Tall shrub	Winner	No change
Myrica gale	11986400	217.677	Tall shrub	Winner	-
Phyllodoce caerulea	11849400	165.0955	Dwarf shrub	Winner	No change
Rhododendron lapponicum	11052300	132.5375	Tall shrub	Winner	No change
Cassiope tetragona	9328900	143.259	Low shrub	Winner	Loser
Ribes nigrum	7761350	72.157	Tall shrub	Winner	-
Ribes spicatum	7063100	58.4415	Tall shrub	Winner	-
Sibbaldia procumbens	6315250	67.4995	Dwarf shrub	Winner	Winner
Arctous alpina	5677050	59.428	Low shrub	Winner	No change
Harrimanella hypnoides	4009550	156.2445	Dwarf shrub	Winner	Loser
Arctous rubra	3538550	35.196	Dwarf shrub	Winner	No change
Salix phylicifolia	3513900	41.1555	Tall shrub	Winner	Winner
Salix hastata	3330050	44.316	Tall shrub	Winner	No change
Rosa majalis	3312700	37.914	Tall shrub	Winner	-
Rosa acicularis	3142400	20.7755	Tall shrub	Winner	-

Rubus chamaemorus	3118050	17.9495	Low shrub	Winner	No change
Vaccinium myrtillus	2951100	38.388	Tall shrub	Winner	Winner
Rubus idaeus	1930500	15.604	Tall shrub	Winner	-
Artemisia dracunculus	1863700	20.9325	Tall shrub	No change	-
Salix lanata	1750150	34.965	Tall shrub	Winner	Winner
Salix niphoclada	1205100	50.194	Tall shrub	Winner	No change
Empetrum nigrum	1010150	6.37	Low shrub	No change	Winner
Artemisia gmelinii	999950	140.6995	Tall shrub	Winner	-
Salix barrattiana	787800	35.48	Tall shrub	Winner	-
Andromeda glaucophylla	653300	141.4375	Low shrub	Winner	-
Vaccinium vitis-idaea	621600	3.0915	Dwarf shrub	No change	Winner
Salix myrsinites	607250	16.9995	Tall shrub	Winner	-
Salix lapponum	541550	8.9625	Tall shrub	Winner	-
Calluna vulgaris	513950	11.876	Tall shrub	No change	Winner
Vaccinium myrtilloides	393400	69.9005	Tall shrub	Winner	-
Salix phlebophylla	278250	10.5635	Dwarf shrub	Winner	No change
Dryas octopetala	-67950	-0.6965	Dwarf shrub	Loser	Loser
Andromeda polifolia	-189600	-1.6365	Tall shrub	Loser	No change
Cornus canadensis	-217500	-3.2755	Low shrub	Loser	-
Thymus praecox	-247250	-20.751	Dwarf shrub	Loser	No change
Salix vestita	-363300	-100	Tall shrub	Loser	-
Salix argyrocarpa	-520100	-90.8635	Tall shrub	Loser	-
Salix glauca	-561000	-10.2125	Tall shrub	No change	Loser

Salix herbacea	-617850	-14.337	Dwarf shrub	Loser	Loser
Vaccinium oxycoccos	-692050	-9.413	Dwarf shrub	No change	-
Betula glandulosa	-805450	-10.4115	Tall shrub	Loser	Loser
Betula nana	-821650	-8.2445	Tall shrub	No change	Winner
Salix rotundifolia	-832450	-30.358	Dwarf shrub	Loser	No change
Vaccinium uliginosum	-1124700	-5.803	Tall shrub	No change	No change
Vaccinium caespitosum	-1299250	-43.703	Tall shrub	Loser	-
Salix reticulata	-1511250	-14.519	Dwarf shrub	Loser	Winner
Loiseleuria procumbens	-1756650	-29.016	Low shrub	Loser	No change
Thymus serpyllum	-1816600	-69.3305	Dwarf shrub	Loser	-
Diapensia lapponica	-1893050	-24.9935	Dwarf shrub	Loser	No change
Salix arctica	-1970200	-38.3465	Low shrub	Loser	No change
Salix polaris	-2066100	-42.7455	Dwarf shrub	Loser	Loser
Betula humilis	-2102700	-53.632	Tall shrub	Loser	-
Salix pulchra	-2785450	-50.1415	Tall shrub	Loser	No change
Artemisia frigida	-2944800	-35.6365	Low shrub	Loser	-
Ribes glandulosum	-3215150	-61.165	Low shrub	Loser	-
Chamaedaphne calyculata	-3364600	-26.015	Tall shrub	Loser	-
Salix rosmarinifolia	-3811250	-71.435	Tall shrub	Loser	-
Arctostaphylos uva-ursi	-4791200	-56.857	Dwarf shrub	Loser	Loser
Dryas integrifolia	-4847350	-76.7265	Dwarf shrub	Loser	No change
Cornus sericea	-4875550	-92.068	Tall shrub	Loser	-

Linnaea borealis	-7510050	-50.1595	Dwarf shrub	Loser	-

Supplementary references

1. Bay, C. *A phytogeographical study of the vascular plants of northern Greenland - north of 74° northern latitude.* (Kommissionen for Videnskabelige Undersøgelser i Grønland, 1992).
2. Feilberg, J. *A phytogeographical study of South Greenland. Vascular plants.* (Kommissionen for Videnskabelige Undersøgelser i Grønland, 1984).
3. Fredskild, B. *A phytogeographical study of the vascular plants of West Greenland (62°20' - 74°00'N).* (Kommissionen for Videnskabelige Undersøgelser i Grønland, 1996).
4. Hultén, E. *Flora of Alaska and the Neighboring Territories.* (Stanford University Press, 1968).
5. Hultén, E. & Fries, M. *Atlas of North European Vascular Plants North of the Tropic of Cancer*. vol. vol 1-3 (Koeltz Scientific Books, 1986).
6. Normand, S. *et al.* A greener Greenland? Climatic potential and long-term constraints on future expansions of trees and shrubs. *Philosophical Transactions of the Royal Society B: Biological Sciences* **368**, 20120479 (2013).
7. Merow, C., Smith, M. J. & Silander, J. A. A practical guide to MaxEnt for modeling species' distributions: what it does, and why inputs and settings matter. *Ecography* **36**, 1058–1069 (2013).
8. Hijmans, R. J., Cameron, S. E., Parra, J. L., Jones, P. G. & Jarvis, A. Very high resolution interpolated climate surfaces for global land areas. *International Journal of Climatology* **25**, 1965–1978 (2005).
9. Ramírez Villegas, J. & Jarvis, A. *Downscaling Global Circulation Model Outputs: The Delta Method Decision and Policy Analysis Working Paper No. 1*. https://cgspace.cgiar.org/handle/10568/90731 (2010).
10. Kattge, J. *et al.* TRY – A Global Database of Plant Traits. *Global Change Biology* (2011) doi:10.1111/j.1365-2486.2011.02451.x.
11. Tamme, R. *et al.* Predicting species' maximum dispersal distances from simple plant traits. *Ecology* **95**, 505–513 (2014).
12. Araújo, M. B. & New, M. Ensemble forecasting of species distributions. *Trends in Ecology & Evolution* **22**, 42–47 (2007).
13. Thuiller, W., Lafourcade, B., Engler, R. & Araújo, M. B. BIOMOD – a platform for ensemble forecasting of species distributions. *Ecography* **32**, 369–373 (2009).

14. R Core Team. R: A language and environment for statistical computing. (2015).
15. Bjorkman, A. D. *et al.* Plant functional trait change across a warming tundra biome. *Nature* **562**, 57 (2018).
