# Supplementary material for: Plant traits poorly predict winner and loser shrub species in a warming tundra biome
Source: Nat Commun. 2023 Jun 28;14:3837. doi: 10.1038/s41467-023-39573-4 (PMC10307830; doi:10.1038/s41467-023-39573-4)
Supplement: Supplementary file 3 — Description of Additional Supplementary Files [file 41467_2023_39573_MOESM3_ESM.pdf]

Description of Additional Supplementary Files

File Name: **Supplementary Data 1**

Description: Summary of all Bayesian models fitted in this study. CI means credible intervals. Parameters in bold are those whose estimates and credible intervals do not overlap zero, and those parameters in categorical variables which are different to each other. All data distributions are Gaussian. No random effects were included in these models. Model priors were non-informative.

File Name: **Supplementary Data 2**

Description: Summary of seed mass records per species. The table contains a comparative of trait metrics calculated using all available trait records per species, and only a sample of five records per species. Numb. = number, Log = logarithmic transformation, MTV = median trait value, SD = standard deviation, COV = coefficient of variation (calculated as SD/mean).

File Name: **Supplementary Data 3**

Description: Summary of SLA records per species. The table contains a comparative of trait metrics calculated using all available trait records per species, and only a sample of five records per species. Numb. = number, Log = logarithmic transformation, MTV = median trait value, SD = standard deviation, COV = coefficient of variation (calculated as SD/mean).

File Name: **Supplementary Data 4**

Description: Summary of plant height records per species. The table contains a comparative of trait metrics calculated using all available trait records per species, and only a sample of five records per species. Numb. = number, Log = logarithmic transformation, MTV = median trait value, SD = standard deviation, COV = coefficient of variation (calculated as SD/mean).
