# Supplementary material for: Plant traits poorly predict winner and loser shrub species in a warming tundra biome
Source: Nat Commun. 2023 Jun 28;14:3837. doi: 10.1038/s41467-023-39573-4 (PMC10307830; doi:10.1038/s41467-023-39573-4)
Supplement: Supplementary file 8 — Reporting Summary [file 41467_2023_39573_MOESM8_ESM.pdf]

nature portfolio

Corresponding author(s): Mariana García Criado

Last updated by author(s): May 29, 2023

Reporting Summary

Nature Portfolio wishes to improve the reproducibility of the work that we publish. This form provides structure for consistency and transparency in reporting. For further information on Nature Portfolio policies, see our Editorial Policies and the Editorial Policy Checklist.

Statistics

For all statistical analyses, confirm that the following items are present in the figure legend, table legend, main text, or Methods section.

n/a	Confirmed	
☐	☒	The exact sample size (*n*) for each experimental group/condition, given as a discrete number and unit of measurement
☐	☒	A statement on whether measurements were taken from distinct samples or whether the same sample was measured repeatedly
☐	☒	The statistical test(s) used AND whether they are one- or two-sided<br>*Only common tests should be described solely by name; describe more complex techniques in the Methods section.*
☐	☒	A description of all covariates tested
☐	☒	A description of any assumptions or corrections, such as tests of normality and adjustment for multiple comparisons
☐	☒	A full description of the statistical parameters including central tendency (e.g. means) or other basic estimates (e.g. regression coefficient) AND variation (e.g. standard deviation) or associated estimates of uncertainty (e.g. confidence intervals)
☐	☒	For null hypothesis testing, the test statistic (e.g. *F*, *t*, *r*) with confidence intervals, effect sizes, degrees of freedom and *P* value noted<br>*Give P values as exact values whenever suitable.*
☐	☒	For Bayesian analysis, information on the choice of priors and Markov chain Monte Carlo settings
☒	☐	For hierarchical and complex designs, identification of the appropriate level for tests and full reporting of outcomes
☒	☐	Estimates of effect sizes (e.g. Cohen's *d*, Pearson's *r*), indicating how they were calculated

Our web collection on statistics for biologists contains articles on many of the points above.

Software and code

Policy information about availability of computer code

Data collection	No specific software was used to collect data.
Data analysis	We used the software and programming language R versions 3.6.2, 3.2.3 and 3.2.0 (R Core Team, 2020) for all analyses. We fitted all Bayesian models using the ‘brms’ package v.2.17.0 (Bürkner, 2017). The species distribution models were fitted with the 'biomod2' package version 2 3.1-73-05 (Thuiller et al. 2009). The 'stats' v.4.2.0 package (R Core Team 2022) was used to perform Principal Component Analyses, the 'AMR' package v.1.8.2 (Berends et al. 2021) to visualize trait space, the RVAideMemoire’ package v.0.9-81-2 (Hervé 2020) to perform pairwise comparisons and the 'vegan' package v.2.6-2 (Oksanen et al. 2020) to calculate distances to centroid.<br><br>Code used for this manuscript is freely available at https://zenodo.org/record/7974602.

For manuscripts utilizing custom algorithms or software that are central to the research but not yet described in published literature, software must be made available to editors and reviewers. We strongly encourage code deposition in a community repository (e.g. GitHub). See the Nature Portfolio guidelines for submitting code & software for further information.

Data

Policy information about availability of data

All manuscripts must include a data availability statement. This statement should provide the following information, where applicable:

- Accession codes, unique identifiers, or web links for publicly available datasets
- A description of any restrictions on data availability
- For clinical datasets or third party data, please ensure that the statement adheres to our policy

Trait data are available at https://www.try-db.org/TryWeb/Home.php (TRY) and https://tundratraitteam.github.io/ (TTT). Cover change over time data will be published at https://github.com/annebj/ITEX30_VegComp. A previous version of this dataset can be accessed at http://polardata.ca/, CCIN Reference Number 10786. Species range data are available as a summarised dataset, together with the rest of input data necessary to reproduce figures and analyses, at https://zenodo.org/record/7974602. DOI for this dataset is 10.5281/zenodo.7974602.

Human research participants

Policy information about studies involving human research participants and Sex and Gender in Research.

Reporting on sex and gender	Not applicable.
Population characteristics	Not applicable.
Recruitment	Not applicable.
Ethics oversight	Not applicable.

Note that full information on the approval of the study protocol must also be provided in the manuscript.

Field-specific reporting

Please select the one below that is the best fit for your research. If you are not sure, read the appropriate sections before making your selection.

☐ Life sciences ☐ Behavioural & social sciences ☒ Ecological, evolutionary & environmental sciences

For a reference copy of the document with all sections, see nature.com/documents/nr-reporting-summary-flat.pdf

Ecological, evolutionary & environmental sciences study design

All studies must disclose on these points even when the disclosure is negative.

Study description	This study investigates the relationship between current range sizes, projected range shifts and past abundance change (response variables) and mean trait values and intraspecific trait variation (effect variables) in tundra shrubs. The majority of models are Bayesian weighted linear regressions based on a calculated index dependent on the number of trait records per species (the structural unit), with a Gaussian data distribution and no random effects. Sample size was dependent on the trait considered, and these are all specified in Supplementary Data 1 together with model structure and outputs for each statistical analysis.
Research sample	We extracted a total of 17,921 trait records from the TRY 5.0 (Kattge et al., 2020) and the Tundra Trait Team (TTT) databases (Bjorkman et al., 2018) for three plant size and economics traits related to competitive ability and dispersal (plant height, SLA and seed mass) for 62 shrub species across three continents. We used projected current range sizes and range shifts data for the 62 shrub species under 24 different climatic scenarios. The past abundance change data was derived from the International Tundra Experiment (ITEX) network for 34 species.
Sampling strategy	We calculated trait values and variation for species that had a minimum of five trait records. We used all available data for every trait that complied with our criteria in order to appropriately capture mean trait values and intraspecific trait variation. We also compared trait values and variation by using all available records and by taking only a random sample of five trait values per species. Both strategies yielded similar metrics.
Data collection	Mariana García Criado and Anne Bjorkman downloaded, curated and cleaned the trait data from TRY and TTT. Anne Blach-Overgaard and Signe Normand quantified and provided the Species Distribution Models-derived data. Anne Bjorkman curated and calculated the ITEX abundance data.
Timing and spatial scale	TRY and TTT data were downloaded in February 2020. They contain data recorded over the last few decades. The data we extracted was limited to shrub species found in the Arctic and with trait records north of 30 degrees latitude. Trait records were recorded from North America, Europe and Asia. ITEX data were recorded between 1970 and 2010 across the same continents.
Data exclusions	Trait data downloaded from TRY and TTT were excluded from the study when i) the values were greater than four standard deviations from each species mean, ii) species had fewer than five trait records, iii) records were from below 30 degrees latitude, iv)

they belonged to experimental manipulations (e.g., warming chambers), v) they were calculated from herbarium specimens, vi) they were calculated as site means instead of single values or individual means.

Reproducibility	Code and data are publicly available at https://zenodo.org/record/7974602.
Randomization	We calculated trait values and variation by using all available records and by taking only a random sample of five trait values per species. Since both strategies yielded similar metrics, we used all available records in order to most appropriately capture mean trait values and intraspecific trait variation.
Blinding	Blinding was not applicable to the study as no participants were involved and no comparison was made between control and experimental studies.

Did the study involve field work? ☐ Yes ☒ No

Reporting for specific materials, systems and methods

We require information from authors about some types of materials, experimental systems and methods used in many studies. Here, indicate whether each material, system or method listed is relevant to your study. If you are not sure if a list item applies to your research, read the appropriate section before selecting a response.

Materials & experimental systems

n/a	Involved in the study
☒	☐ Antibodies
☒	☐ Eukaryotic cell lines
☒	☐ Palaeontology and archaeology
☒	☐ Animals and other organisms
☒	☐ Clinical data
☒	☐ Dual use research of concern

Methods

n/a	Involved in the study
☒	☐ ChIP-seq
☒	☐ Flow cytometry
☒	☐ MRI-based neuroimaging
